# Supplementary material for: A robust activity marking system for exploring active neuronal ensembles
Source: eLife. 2016 Sep 23;5:e13918. doi: 10.7554/eLife.13918 (PMC5035142; doi:10.7554/eLife.13918)
Supplement: Supplementary file 1. — See ‘Methods’ for further details. 10mers used in PRAM and enhancer elements E1-E3 (Figure 1a, and Figure 1—figure supplement 2b) are highlighted in bold. 10mers with corrected p-value≥0.05 are marked in grey color. DOI: http://dx.doi.org/10.7554/eLife.13918.023 [file elife-13918-supp1.docx]

**SUPPLEMENTARY FILE 1**

A full list of AP-1 containing 10mers (128 in total) ranked by their enrichment factor. See ‘Methods’ for further details. 10mers used in P_RAM_ and enhancer elements E1-E3 (**Figure 1a**, and **Figure 1-figure supplement 2b**) are highlighted in bold. 10mers with corrected p-value ≥ 0.05 are marked in grey color.

| **10mers** | **Enrichment factor** |
| --- | --- |
| CGGTGACTCA | 44 |
| CGATGACTCA | 22 |
| TGACTCATCG | 18.67 |
| CCGTGACTCA | 16 |
| CGCTGACTCA | 14.67 |
| TGACTCACGC | 11.2 |
| ACGTGACTCA | 10.67 |
| **TGACTCATTA** (**E2**) | 10.29 |
| **TGACTCACCC (E1)** | 10 |
| TGACTCACGG | 9.5 |
| TGACTCAGCG | 9.33 |
| TGACTCATTG | 9.14 |
| GAGTGACTCA | 8.95 |
| TGACTCATGC | 8.57 |
| CTGTGACTCA | 8.38 |
| **GTATGACTCA** (**E3**) | 8.36 |
| TGACTCACCG | 8 |
| GCGTGACTCA | 8 |
| TGACTCATGA | 8 |
| TGATGACTCA | 8 |
| ATCTGACTCA | 7.73 |
| **TCGTGACTCA** (**P_RAM_**) | 7.55 |
| GGGTGACTCA | 7.33 |
| TGACTCACTC | 7.27 |
| TGACTCATCT | 7.16 |
| TGACTCACGA | 7 |
| TGACTCACAT | 7 |
| TGACTCAGCC | 6.96 |
| TGACTCACAC | 6.92 |
| AAATGACTCA | 6.79 |
| CTATGACTCA | 6.67 |
| TTATGACTCA | 6.67 |
| TGACTCACCA | 6.67 |
| TGACTCATCC | 6.67 |
| TGACTCACTG | 6.63 |
| CATTGACTCA | 6.57 |
| TGACTCATGG | 6.53 |
| CAATGACTCA | 6.32 |
| TGACTCATTC | 6.17 |
| TGACTCATAC | 6.12 |
| TGACTCACAG | 5.96 |
| CAGTGACTCA | 5.9 |
| TGTTGACTCA | 5.85 |
| GTCTGACTCA | 5.8 |
| TGACTCAATA | 5.78 |
| GATTGACTCA | 5.71 |
| AAGTGACTCA | 5.7 |
| TGACTCACTT | 5.6 |
| ATGTGACTCA | 5.54 |
| TGACTCATGT | 5.5 |
| CACTGACTCA | 5.44 |
| TGACTCAGAT | 5.44 |
| GTGTGACTCA | 5.43 |
| TGACTCAACG | 5.33 |
| TGACTCAAGC | 5.33 |
| ATATGACTCA | 5.25 |
| TGACTCATTT | 5.25 |
| TGGTGACTCA | 5.22 |
| TGACTCATAA | 5.2 |
| CTCTGACTCA | 5.16 |
| TGACTCACGT | 5.14 |
| TGACTCATCA | 5.04 |
| GAATGACTCA | 5.04 |
| AGATGACTCA | 5.04 |
| TAATGACTCA | 5 |
| GGCTGACTCA | 5 |
| ACATGACTCA | 5 |
| TGACTCATAT | 4.94 |
| GACTGACTCA | 4.89 |
| TGACTCATAG | 4.75 |
| CCATGACTCA | 4.69 |
| TGACTCAGCA | 4.67 |
| GCATGACTCA | 4.63 |
| TTCTGACTCA | 4.57 |
| TGACTCAGTC | 4.38 |
| TTGTGACTCA | 4.38 |
| TGACTCAGTG | 4.36 |
| AGCTGACTCA | 4.21 |
| TGCTGACTCA | 4.11 |
| CGTTGACTCA | 4 |
| AGTTGACTCA | 4 |
| TGACTCAATT | 4 |
| GCCTGACTCA | 4 |
| TGACTCAGAG | 3.95 |
| TGACTCAGGC | 3.87 |
| TGACTCAAGG | 3.78 |
| TGACTCAATG | 3.71 |
| TGACTCAGTA | 3.68 |
| TGACTCACTA | 3.62 |
| GGATGACTCA | 3.48 |
| TAGTGACTCA | 3.48 |
| TGACTCAGAA | 3.46 |
| CTTTGACTCA | 3.43 |
| TGACTCAGAC | 3.41 |
| TGACTCAGTT | 3.36 |
| TGACTCAACC | 3.27 |
| TACTGACTCA | 3.25 |
| TCATGACTCA | 3.2 |
| AATTGACTCA | 3.06 |
| TGACTCAAAG | 3.04 |
| TGACTCAAGT | 3 |
| GTTTGACTCA | 2.95 |
| TATTGACTCA | 2.89 |
| TGACTCAACT | 2.89 |
| TGACTCAATC | 2.89 |
| GCTTGACTCA | 2.75 |
| TGACTCAACA | 2.74 |
| TGACTCAAAC | 2.72 |
| GGTTGACTCA | 2.67 |
| TGACTCAGGG | 2.53 |
| TCCTGACTCA | 2.48 |
| AGGTGACTCA | 2.38 |
| TGACTCAAAT | 2.33 |
| TTTTGACTCA | 2.27 |
| ATTTGACTCA | 2.22 |
| AACTGACTCA | 2.21 |
| TGACTCAGGA | 2.1 |
| TCTTGACTCA | 2 |
| CCCTGACTCA | 1.9 |
| TGACTCACCT | 1.74 |
| TGACTCAAAA | 1.69 |
| ACCTGACTCA | 1.68 |
| TGACTCAAGA | 1.33 |
| ACTTGACTCA | 1.18 |
| TGACTCAGGT | 1.11 |
| CCTTGACTCA | 0.57 |
