# Supplementary material for: A robust activity marking system for exploring active neuronal ensembles
Source: eLife. 2016 Sep 23;5:e13918. doi: 10.7554/eLife.13918 (PMC5035142; doi:10.7554/eLife.13918)
Supplement: Supplementary file 2. — DOI: http://dx.doi.org/10.7554/eLife.13918.024 [file elife-13918-supp2.docx]

**SUPPLEMENTARY FILE 2**

Experimental Conditions and Statistics

| **Figure** | **Experimental Conditions** | **Statistical Test** |
| --- | --- | --- |
| **1a** | Cultured cells were transfected on DIV5 and stimulated with KCl (35 mM) for 6 hrs on DIV8. TTX and APV were applied 1 hr prior to stimulation. | *F*(4,25) = 7.57, One-way ANOVA, Tukey’s post-hoc test. |
| **1b** | Cultured cells were transfected on DIV5 and stimulated with KCl (35 mM) for 6 hrs on DIV7. TTX and APV were applied 1 hr prior to stimulation. | *F*(2,17) = 6.92, One-way ANOVA, Tukey’s post-hoc test. |
| **1c** | Cultured cells were transfected on DIV5 and stimulated with KCl (35 mM) for 6 hrs on DIV7-8. TTX and APV were applied 1 hr prior to stimulation. | P_RAM_ vs. cFos, *t*(15) = 2.80; P_RAM_ vs. Npas4, *t*(12) = 0.73; P_RAM_ vs. BDNF, *t*(12) = 1.27; P_RAM_ vs. CREB, *t*(14) = 2.58; P_RAM_ vs. MEF2, *t*(14) = 2.81. Student’s t-tests. |
| **1d** | Same as in **Figure 1b**. | P_RAM_ No stim vs. P_RAM_ KCl: *t*(14) = 3.53; P_RAM_ No stim vs. ESARE No stim: *t*(12) = 3.29; ESARE No stim. vs. ESARE KCl: *t*(10) = 3.24; P_RAM_ Fold vs. ESARE Fold: *t*(12) = 3.44, Student’s t-tests. |
| **1e** | Cultured cells were transfected on DIV5 and harvest on DIV8 following incubation with TTX and APV for 7 hrs. | *F*(3,36) = 8.44, One-way ANOVA, Dunnett’s post-hoc test. |
| **1g** | Cultured cells were transfected on DIV5 and doxycycline was added at the same time. Cultures were stimulated with KCl (17.5 mM) for 6 hrs on DIV8. TTX was added the night before stimulation. Doxycycline was removed prior to stimulation by washing the wells twice with conditional media. | –Dox/d2tTA vs. -Dox/tTA, *t*(4) = 7.40; –Dox/d2tTA vs. +Dox/d2tTA, *t*(4) = 8.42; –Dox/tTA vs. +Dox/tTA, *t*(4) = 4.64; +Dox/d2tTA vs. +Dox/tTA, *t*(4) = 3.00, Student’s t-tests. |
| **1h** | AAV-RAM-tdTomato virus was added to cultures on DIV7 and immunocytochemistry was performed on DIV14. Cultures were stimulated overnight on DIV13 with bicuculline and 4AP. Doxycycline was added on DIV7. |  |
| **1i** | Same as in **Figure 1h**. | *F*(2,6) = 2521, One-way ANOVA, Tukey’s post-hoc test. |
| **2c** |  | *F*(2,12) = 54.95. One-way ANOVA, Tukey’s post-hoc test. |
| **2f** |  | Effect of time: *F*(3,22) *=* 66.03, Effect of detection method (mKate2 vs α-mKate2): *F*(3,22) *= 0.*66, Two-way ANOVA, Bonferroni post-hoc test. |
| **3c** |  | *F*(3,21) = 36.56. One-way ANOVA, Tukey’s post-hoc test. |
| **4g** |  | *t*(7) = 10.04, Student’s t-test. |
| **4h** |  | *t*(7) = 2.79, Student’s t-test. |
| **4i** |  | *t*(7) = 3.22, Student’s t-test. |
| **5c** |  | *F*(3,14) = 31.73, One-way ANOVA, Tukey’s post-hoc test. |
| **5e** |  | *F*(3,11) = 17.22, One-way ANOVA, Tukey’s post-hoc test. |
| **5h** |  | *t*(4) = 6.08, Student’s t-test. |
| **6c** |  | *t*(5) = 4.04, Student’s t-test. |
| **6f** |  | RAM-luc;UASflp NO HS vs. HS *t*(84) = 8.66, UAS-flp;RAM-luc NO HS vs. HS *t*(90) = 8.89, Student's t-tests. |
| **6h** |  | *t*(45) = 3.13, Student's t-test. |
| **7d** |  | *t*(5) = 2.44, Student’s t-test. |
| **7f** | Cultured cells were infected on DIV7 and stimulated with KCl for 6 hrs on DIV13. TTX, APV and Nimodipine were applied 1 hr prior to stimulation. | *F*(3,8) = 57.96. One-way ANOVA, Tukey’s post-hoc test. |
| **7g** | Same as in **Figure 7f**, but stimulated with various neurotrophic factors and drugs | TTX / APV vs. +KCl, *t*(4) = 6.71; TTX / APV vs. +BDNF, *t*(4) = 6.20; TTX / APV vs. +NT3, *t*(4) = 8.62; TTX / APV vs. +NT4, *t*(4) = 14.85; TTX / APV vs. +IGF1, *t*(4) = 1.02; TTX / APV vs. +EGF *t*(4) = 1.51; TTX / APV vs. +Forskolin, *t*(4) = 2.30; TTX / APV vs. +PMA, *t*(4) = 16.02, Student’s t-tests. |
| **1-figure supplement 2a** | Cultured cells were transfected on DIV5 and stimulated with KCl (35 mM) for 6 hrs on DIV12. TTX and APV were applied 1 hr prior to stimulation. | *t*(12) = 2.93, Student’s t-test. |
| **1-figure supplement 2b** | Same as in **Figure 1a**. | *F*(4,25) = 13.02, One-way ANOVA, Tukey post-hoc test. |
| **1-figure supplement 2c** | Same as in **Figure 1**-**figure supplement 2a**. | *F*(4,29) = 7.18, One-way ANOVA, Dunnett’s post-hoc test. |
| **1-figure supplement 2d** | Same as in **Figure 1**-**figure supplement 2a**. | *F*(3,27) = 4.36, One-way ANOVA, Dunnett’s post-hoc test. |
| **1-figure supplement 3a** | Same as in **Figure 1c**. | P_RAM_ No Stim vs. MEF2 No Stim, *t*(14) = 2.36, P_RAM_ KCl vs. cFos KCl, *t*(15) = 2.41, P_RAM_ KCl vs. CREB KCl, *t*(14) = 2.87, P_RAM_ KCl vs. MEF2 KCl, *t*(14) = 3.00, Student’s t-tests. All other comparisons are non-significant. |
| **1-figure supplement. 3b** | Cultured cells were transfected on DIV5 and stimulated at DIV12 for 6 hrs with bicuculline. Nimodipine and APV were applied 1 hr prior to stimulation. | *F*(4,15) = 8.68, One-way ANOVA, Tukey’s post-hoc test. |
| **1-figure supplement 3c** | Cultured cells were transfected on DIV5 and stimulated on DIV8 with various growth factors and pharmacological agents.  TTX and APV were applied 1 hr prior to stimulation for all conditions. | TTX / APV vs. +BDNF, *t*(6) = 9.98; TTX / APV vs. +NT3, *t*(6) = 7.95; TTX / APV vs. +NT4, *t*(6) = 5.32; TTX / APV vs. +IGF1, *t*(6) = 0.47; TTX / APV vs. +EGF, *t*(6) = 1.34; TTX / APV vs. +Forskolin, *t*(6) = 2.73; TTX / APV vs. +PMA, *t*(6) = 2.68. TTX / APV vs. +DMSO, *t*(6) = 0.92, Student’s t-tests. |
| **1-figure supplement 3d** | Glia cultures were transfected at DIV14 and stimulated with KCl at DIV17. TTX and APV were applied 1 hr prior to stimulation. | *t*(2) = 0.03, Student’s t-test. |
| **1-figure supplement 3e** | Same as in **Figure 1g**. | No Stim conditions: –Dox/d2tTA vs. -Dox/tTA, *t*(4) = 17.74; +Dox/d2tTA vs. +Dox/tTA, *t*(4) = 1.64; KCl conditions: –Dox/d2tTA vs. -Dox/tTA, *t*(4) = 5.96; +Dox/d2tTA vs. +Dox/tTA, *t*(4) = 2.33, Student’s t-tests. |
| **1-figure supplement 4a** | Cultured cells were infected on DIV7 and quantified on DIV14. The day before quantification, cultures were stimulated overnight with bicuculline and 4AP. Doxycycline was not added. |  |
| **1-figure supplement 4b** | Same as in **Figure 1-figure supplement 4a**. |  |
| **2-figure supplement 1c** |  | *F*(2,7) = 4.20. One-way ANOVA, Tukey’s post-hoc test. |
| **3-figure supplement 2d** |  | *t*(6) = 28.51, Student’s t-test. |
| **3-figure supplement 3c** |  | *F*(3,12) = 41.99. One-way ANOVA, Tukey’s post-hoc test. |
| **7-figure supplement 1c** |  | *t*(5) = 3.70, Student’s t-test. |
| **7-figure supplement 2c** |  | *t*(6) = 1.71, Student’s t-test. |
| **7-figure supplement 4a** |  | V1: Effect of treatment: *F*(1,12) *=* 100.5, p<0.0001; Effect of cell type: *F*(1,12) *=* 62.09, p<0.0001;  Interaction: *F*(1,12) *=* 35.11, p<0.0001, Two-way ANOVA, Tukey’s post-hoc test.  DG: Effect of treatment: *F*(1,20) *=* 325.2, p<0.0001; Effect of cell type: *F*(1,20) *=* 57.05, p<0.0001;  Interaction: *F*(1,20) *=* 41.72, p<0.0001, Two-way ANOVA, Tukey’s post-hoc test. |
